# Chapped Hands

**Published:** 1858-12

**Authors:** 


### CHAPPED HANDS.

THIS is an annoyance in winter-time; while to keep them soft and white is sometimes very desirable—to do this, wash the hands not more than once or twice a day, and always in water a little warm, using the finest, purest white soap. Rinse them well, so that the soap shall be entirely removed, then wipe them with a soft, dry towel, closing the operation by rubbing the hands with one another very freely until there is a feeling of comfortable softness in them.

At bed-time, especially of the coldest days, a few drops of sweet-oil should be most thoroughly rubbed with one hand into the other. If coal must be handled, or fires made or replenished, do not go near the fire until a pair of gloves, lined with some soft material are put on.

